# The Marine-Derived Macrolactone Mandelalide A Is an Indirect Activator of AMPK

**DOI:** 10.3390/md20070418

**Published:** 2022-06-27

**Authors:** Daphne R. Mattos, Xuemei Wan, Jeffrey D. Serrill, Minh H. Nguyen, Ian R. Humphreys, Benoit Viollet, Amos B. Smith, Kerry L. McPhail, Jane E. Ishmael

**Affiliations:** 1 Department of Pharmaceutical Sciences, College of Pharmacy, Corvallis, OR 97331, USA; mattosd@oregonstate.edu (D.R.M.); wanxuemei0109@gmail.com (X.W.); jeffrey.serrill@gmail.com (J.D.S.); ianhumph@uw.edu (I.R.H.); kerry.mcphail@oregonstate.edu (K.L.M.); 2 Department of Chemistry, Laboratory for Research on the Structure of Matter, and Monell Chemical Senses Center, University of Pennsylvania, Philadelphia, PA 19104, USA; mnguyen@incyte.com (M.H.N.); smithab@sas.upenn.edu (A.B.S.III); 3 CNRS, INSERM, Institut Cochin, Université Paris Cité, F-75014 Paris, France; benoit.viollet@inserm.fr

**Keywords:** OXPHOS, polyketide, ATP synthase, ATPase, oxidative phosphorylation, AMP-activated protein kinase, macrolactone

## Abstract

The mandelalides are complex macrolactone natural products with distinct macrocycle motifs and a bioactivity profile that is heavily influenced by compound glycosylation. Mandelalides A and B are direct inhibitors of mitochondrial ATP synthase (complex V) and therefore more toxic to mammalian cells with an oxidative metabolic phenotype. To provide further insight into the pharmacology of the mandelalides, we studied the AMP-activated protein kinase (AMPK) energy stress pathway and report that mandelalide A is an indirect activator of AMPK. Wild-type mouse embryonic fibroblasts (MEFs) and representative human non-small cell lung cancer (NSCLC) cells showed statistically significant increases in phospho-AMPK (Thr172) and phospho-ACC (Ser79) in response to mandelalide A. Mandelalide L, which also harbors an A-type macrocycle, induced similar increases in phospho-AMPK (Thr172) and phospho-ACC (Ser79) in U87-MG glioblastoma cells. In contrast, MEFs co-treated with an AMPK inhibitor (dorsomorphin), AMPKα-null MEFs, or NSCLC cells lacking liver kinase B1 (LKB1) lacked this activity. Mandelalide A was significantly more cytotoxic to AMPKα-null MEFs than wild-type cells, suggesting that AMPK activation serves as a protective response to mandelalide-induced depletion of cellular ATP. However, LKB1 status alone was not predictive of the antiproliferative effects of mandelalide A against NSCLC cells. When EGFR status was considered, erlotinib and mandelalide A showed strong cytotoxic synergy in combination against erlotinib-resistant 11-18 NSCLC cells but not against erlotinib-sensitive PC-9 cells. Finally, prolonged exposures rendered mandelalide A, a potent and efficacious cytotoxin, against a panel of human glioblastoma cell types regardless of the underlying metabolic phenotype of the cell. These results add biological relevance to the mandelalide series and provide the basis for their further pre-clinical evaluation as ATP synthase inhibitors and secondary activators of AMPK.

## 1. Introduction

The mandelalides (A to L) are a family of polyketide macrolactones originally isolated from a new *Lissocliunum* tunicate species discovered in and around Algoa Bay in the Eastern Cape of South Africa [1,2,3,4]. An extensive metagenomic analysis of the source tunicate subsequently revealed the presence of a symbiotic *Verrucomicrobia* bacterium associated with the zooid fraction of the tunicate from which the proposed biosynthetic gene cluster for the core mandelalide structures was assembled [5]. With recognition that the mandelalide series is produced by an unculturable bacterial symbiont and rare host animal within a unique marine ecosystem [1,5], access to material through organic synthetic chemistry has remained the only feasible way to continue to evaluate the unusual bioactivity profile of these complex natural products [1,4]. Fortunately, total synthesis of the reassigned absolute structure of mandelalide A [2] was achieved in a short amount of time by the research groups of Fürstner [6], Altmann [7], Carter [8] and Smith [9], allowing confirmation of potent nanomolar cytotoxic activity of glycosylated mandelalides A and B in cell-based assays relative to the aglycone mandelalide C [3]. Further testing of synthetic and natural mandelalides against sensitive human cancer cell types revealed a distinct structure-activity relationship and led to a classification of the individual macrolactones on the basis of three distinct macrocycle motifs represented in the known mandelalide (A to L) series [4]. These macrocycles were designated as A-type, with a simple lactone connection (as found in mandelalides A and L), B-type, containing a butyrolactone moiety (found in mandelalides B, I, J and K), and C-type 23-hydroxy butyrolactone-containing macrocyles (found in mandelalides C to H) [4]. In turn, the mechanistic basis of mandelalide action against human cancer cells was also solved for these base structures; mandelalides A and B are site-specific inhibitors of mitochondrial ATP synthase (complex V) and inhibit the aerobic respiratory capacity of mammalian cells with acute or chronic treatment, whereas mandelalide C showed no effect on mitochondrial function in these assays [4]. The observed sensitivity of human cancer cells to the mandelalides can therefore be attributed to several major factors: the basal metabolic phenotype of the cell, the glycosylation status of the mandelalide macrocycle and the ability of the cell to adapt and maintain homeostasis [3,4].

The ability of cancer cells to deregulate their energy metabolism has been recognized for many years and is now considered a hallmark of cancer cell biology [10,11]. This characteristic was first described as the Warburg effect, whereby cancer cells decrease their reliance on oxidative phosphorylation (OXPHOS) and utilize glycolysis even in the presence of oxygen and functional mitochondria [12,13,14,15]. Such metabolic flexibility provides a survival advantage for cancer cells when nutrient demands are high; however, it is now increasingly clear that a preference for aerobic glycolysis over OXPHOS does not generalize to all cancer cells [15,16]. Cancer cells, like normal mammalian cells, have access to numerous signaling pathways for regulation of nutrient uptake and can adapt their metabolism to match the tumor microenvironment [15,16]. Furthermore, pathways for mitochondrial respiration frequently remain intact allowing OXPHOS or aerobic glycolysis to proceed if a particular nutrient source becomes limited [15,17]. Some cancer types also appear to be inherently more dependent on OXPHOS for production of ATP, while factors such as genetic mutation and anticancer treatments can induce upregulation of OXPHOS and dysregulation of ATP synthase subunits [18,19]. Taken together, these findings have inspired new interest in pharmacological inhibitors of OXPHOS and the feasibility of targeting this metabolic pathway as an anticancer treatment [18,19,20,21].

**Figure 1:**
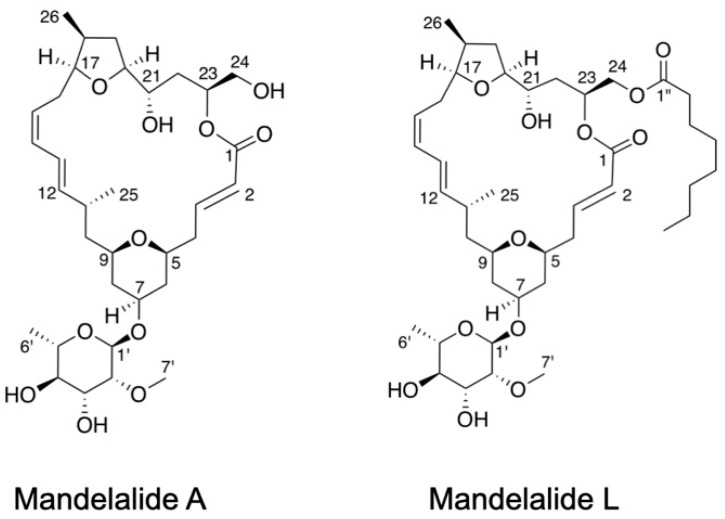
Molecular structures of natural product mandelalides with an A-type macrocycle: mandelalides A and L.

Polyketide macrolactones are just one class of natural products from a variety of sources that have been identified as inhibitors of ATP synthase (reviewed in [22]). Other examples include the oligomycins from *Streptomycin* sp. [23], apoptolidins from *Nocardiopsis* [24], *Amycalotopsis* sp. [25] and ammocidin A from *Saccharothrix* sp. [26]. In our previous study of apoptolidins from soil bacteria isolates originating from a freshwater habitat in Kalimanta, Indonesia, we found apoptolidins A and C to be acute activators of AMP-activated protein kinase (AMPK) [25]. AMPK is the major nutrient sensor in eukaryotic cells and is activated in rapid response to metabolic stress to restore intracellular homeostasis [27]. The apoptolidins induced a unique pattern of adaptive changes in cellular targets that lie downstream of AMPK and signal to drive catabolic processes such as regulation of the lipid metabolism and macroautophagy [25]. Transient activation of AMPK, in response to ATP depletion, was found to be an additional predictor of cancer cell sensitivity to apoptolidin A; representative human glioblastoma and lung cancer cells with an oxidative metabolic phenotype showing robust changes in AMPK stress signaling, whereas more glycolytic cell types lacked this response [25]. Given that the differential sensitivity of human cancer cells to the mandelalide series remains relatively unexplored [4,9], the goal of the present study was to examine the AMPK stress response to cytotoxic mandelalides with an A-type macrocycle. Using genetically-modified mouse embryonic fibroblasts (MEFs), human glioblastoma and human non-small cell lung cancer (NSCLC) cells, we examined the phosphorylation status of key residues in the AMPK signal transduction pathway after acute exposure to mandelalides A and L (Figure 1). We also expanded our analysis of the cytotoxic potential of mandelalide A alone and as a combinatorial treatment in representative NSCLC cells that have a tumor suppressor liver kinase B1 (LKB1) deletion and that harbor clinically-relevant mutations in endothelial growth factor receptor (EGFR).

## 2. Results

### 2.1. Mandelalide A Is an Indirect Activator of AMPK

**Figure 2:**
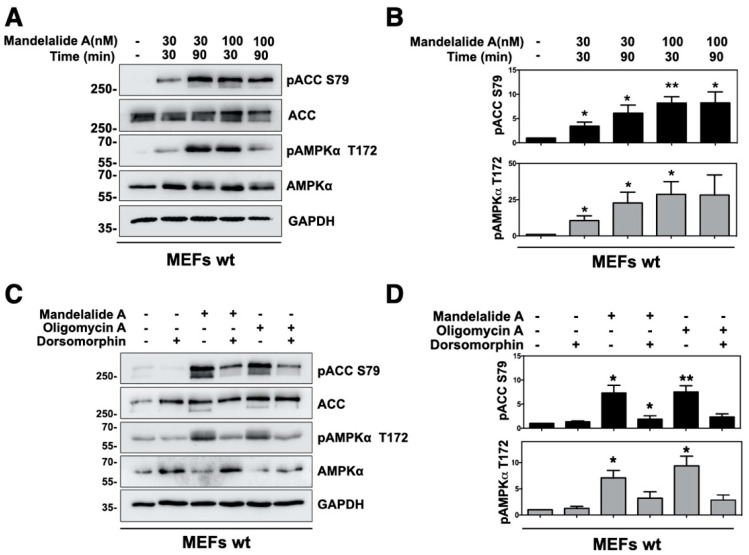
Mandelalide A induces time and concentration-dependent phosphorylation of AMPK and ACC that is suppressed by the AMPK inhibitor dorsomorphin. (**A**) Immunoblot analysis of MEFs treated with mandelalide A (30 nM or 100 nM) for 30 min or 90 min. Whole cell lysates were probed with antibodies for phospho-AMPK, phospho-ACC, total AMPK, total ACC and GAPDH as indicated. Blots are representative of a single experiment that was repeated three times. (**B**) Histograms show the quantification of immunoblot data shown in A from three independent experiments; values represent band intensity of phospho-AMPK/total AMPK and phospho-ACC/total ACC, normalized to GAPDH. (**C**) Immunoblot analysis of MEFs treated with mandelalide A (30 nM) or oligomycin A (1 µM) with, or without, dorsomorphin (10 mM) for 1 h. Whole cell lysates were probed with antibodies as indicated. Blots are representative of a single experiment that was repeated at least three times. (**D**) Histograms show quantification of immunoblot data shown in C; values represent band intensity of the phosphorylated protein, relative to total (p-AMPK/total AMPK and p-ACC/total ACC) normalized to GAPDH. Statistical significance of change relative to vehicle (0.1% DMSO) is indicated in B and D as * *p* < 0.05, ** *p* < 0.01.

As human cancer cells show wide variations in sensitivity to mandelalides [3,4], immortalized MEFs were used as a model cell type to investigate the phosphorylation status of AMPK and a direct downstream target, acetyl CoA carboxylase (ACC), in response to acute exposure to mandelalide A. Wild type MEFs were treated with, or without, mandelalide A (30 nM or 100 nM) for up to 90 min, and whole cell lysates prepared for immunoblot analysis of phospho-AMPKα (Thr172), phospho-ACC (Ser79), total AMPKα, total ACC and the ubiquitously expressed enzyme glyceraldehyde-3-phophate dehydrogenase (GAPDH) as a loading control protein. Treatment with mandelalide A induced time- and concentration-dependent increases in the phosphorylation status of AMPKα (Thr172) and ACC (Ser79), relative to control cells treated with vehicle (0.1% DMSO) alone (Figure 2A,B). Increases in phospho-AMPKα (Thr172) and phospho-ACC (Ser79) were statistically significant at 30 min (30 nM or 100 nM mandelalide A) or 90 min (30 nM mandelalide A) and plateaued, in that the magnitude of change in phospho-AMPKα (Thr172) was not statistically significant at 90 min in response to the higher (100 nM) mandelalide A concentration (Figure 2B). In follow up studies, MEFs were therefore treated for 60 min with either mandelalide A (30 nM), oligomycin A (1 μM), or vehicle (0.1% DMSO) in the presence, or absence, of a direct AMPK inhibitor dorsomorphin (10 µM). Immunoblot analysis of these lysates showed the same basic pattern of AMPK stress signaling in response to mandelalide A or oligomycin A (Figure 2C,D). Both ATP synthase inhibitors induced statistically significant increases in phospho-AMPKα (Thr172) and phospho-ACC (Ser79), and these responses were strongly attenuated in cells co-treated with dorsomorphin (Figure 2C,D). In contrast, MEFs treated with dorsomorphin (10 µM) alone, or vehicle (0.1% DMSO), showed no evidence of AMPK activation (Figure 2A–D).

**Figure 3:**
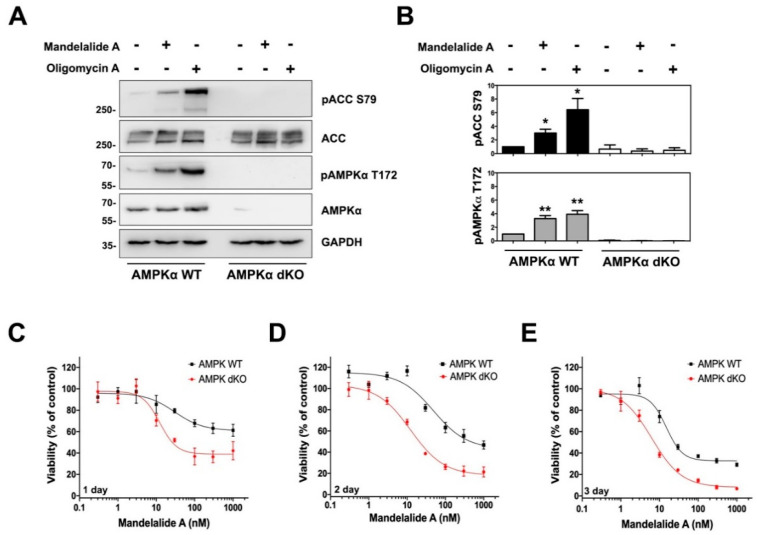
Mandelalide-induced phosphorylation of ACC requires AMPK and confers a survival advantage to wild-type mouse embryonic fibroblasts (MEFs). (**A**) Immunoblot analysis of wild-type and AMPKα-null double knockout (dKO) MEFs treated with mandelalide A (30 nM) or oligomycin A (1 µM) for 90 min. Whole cell lysates were probed with antibodies for phospho-AMPK, phospho-ACC, total AMPK, total ACC and GAPDH as indicated. Blots are representative of a single experiment that was repeated three times (**B**) Histograms show quantification of immunoblot data shown in A from three independent experiments. Values represent band intensity of phospho-AMPK/total AMPK and phospho-ACC/total ACC, normalized to GAPDH. Statistical significance of change relative to vehicle (0.1% DMSO) is indicated as * *p* < 0.05, ** *p* < 0.01. (**C**–**E**) Concentration-dependent change in the viability of AMPKα-null MEFs and wild-type MEFs after 24 h (**C**), 48 h (**D**) and 72 h (**E**). MEFs were continuously exposed to mandelalide A or vehicle (0.1% DMSO) and cell viability determined at the endpoint of the assay using a CellTiter-Glo^®^ assay with the viability of vehicle-treated cells defined as 100%. Graphs represent mean viability ± S.E. (*n* = 3 wells per treatment) and curves represent the fit of data points by nonlinear regression analysis to a logistic equation. Data represent one comparison from at least three independent experiments.

AMPK is a heterotrimeric protein complex composed of an α catalytic subunit and regulatory β and γ subunits [27]. To determine if AMPK activation by mandelalide A is dependent on the catalytic subunit, we utilized AMPKα-null MEFs, lacking both α1 and α2 isoforms of AMPK. Wild-type and double-knockout cells were treated in parallel for 60 min with or without mandelalide A (30 nM) or oligomycin A (1 μM). Immunoblot analysis of these cell lysates revealed statistically significant increases in phospho-AMPKα (Thr172) and phospho-ACC (Ser79) in wild-type MEFs in response to both compounds (Figure 3A,B). In contrast, phosphorylation of ACC (Ser79) was not detected in AMPKα-null MEFs treated with either compound (Figure 3A,B). Taken together, these results show that short-term exposure to mandelalide A is sufficient to activate an AMPK stress response that is dependent on the presence of the AMPKα subunit.

### 2.2. AMPKα Confers a Survival Advantage against Mandelalide A-Induced Cytotoxicity

To study the functional significance of AMPKα activation, wild-type and AMPKα-null MEFs were treated in parallel with increasing concentrations of mandelalide A (0.3 nM to 1 μM) or vehicle (0.1% DMSO) and the metabolic capacity of cells was analysed at each of three different end points (24, 48 or 72 h) as a measure of viability. Mandelalide A induced concentration-dependent decreases in cell viability in all assays, however AMPKα-null cells were consistently more sensitive than wild-type MEFs at each end point (Figure 3C–E). After 24 h, mandelalide A displayed limited cytotoxic efficacy against wild-type MEFs with a relatively modest 40% reduction in cell viability versus a 60% reduction in the viability of AMPKα-null MEFs at the highest concentration tested (1 μM) (Figure 3C). If exposure times were extended to 48 h or 72 h, the efficacy of mandelalide A was greatly enhanced against both wild-type and AMPKα-null MEFs, however the concentration-response curves of AMPKα-null MEFs consistently fell to the left of wild-type cells (Figure 3C–E). Nonlinear regression analysis of these cell viability data revealed that the relative EC_50_ of mandelalide A was lower against AMPKα-null MEFs than wild-type MEFs at both 48 h (13.5 ± 1.4 nM versus 37.1 ± 0.1 nM) and 72 h (10.6 ± 1.9 nM versus 33.0 ± 4.9 nM) (Table 1). These findings indicate that the AMPK complex serves a protective role against mandelalide A-induced cytotoxicity and are consistent with the physiological role of AMPK.

### 2.3. Analysis of Mandelalide A-Induced Changes in AMPK Status and NSCLC Cell Growth as a Function of LKB1 Expression

**Figure 4:**
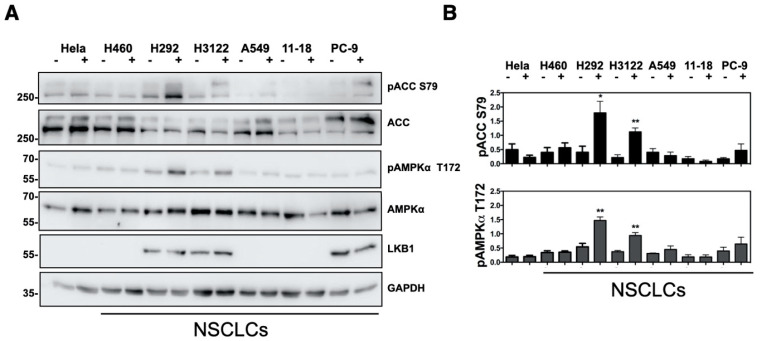
Immunoblot analysis of mandelalide-induced phosphorylation of AMPK and ACC in human cancer cells with differing LKB1 status. (**A**) Immunoblot analysis of AMPK and ACC status in HeLa cervical and a panel of NSCLC cells (NCI-H460, H292, H3122, A549, 11-18 and PC-9) treated with (+) mandelalide A (30 nM) or (−) vehicle (0.1% DMSO) for 1 h. Whole cell lysates were collected, processed for western blot and probed with primary antibodies against phospho-AMPK, phospho-ACC, total AMPK, total ACC, LKB1 and GAPDH, as indicated. (**B**) Histograms show quantification of phospho-AMPK/total AMPK and phospho-ACC/total ACC, normalized to GAPDH from three independent experiments. The statistical significance of change relative to vehicle (0.1% DMSO) is indicated as * *p* < 0.05, ** *p* < 0.01.

Given the early, promising bioactivity profile of mandelalides A and B against LKB1-null NCI-H460 lung cancers cells [1], yet differential sensitivity of cells comprising the NCI-60 lung cancer cell panel [4], we investigated AMPK signaling and growth characteristics of NSCLC cells with differing LKB1 status. LKB1 is frequently deleted or mutated in human NSCLC cells and is considered a clinically significant mutation [28]. Furthermore, LKB1 is a major regulator of AMPK activity and functions as an upstream kinase that phosphorylates AMPKα at the Thr172 position [29]. For these studies, six NSCLC cell lines (NCI-H460, H292, H3122, A549, 11-18 and PC-9), and LKB1-null HeLa cervical cancer cells, were treated with, or without, mandelalide A (30 nM) for 1 h and lysates were collected for immunoblot analysis. As anticipated, a single immunoreactive band corresponding to LKB1 was detected in H292, H3122 and PC-9 lung cancer cell lysates, whereas A549, 11-18 and H460 lung cancer cells and HeLa cervical cancer cells lacked LKB1 expression (Figure 4A). These cancer cell lines had similar basal expression of AMPKα and ACC but showed a differential pattern of phosphorylation in response to mandelalide A treatment (Figure 4A,B). Increases in phospho-AMPKα (Thr172) and phospho-AC (Ser79) were observed in H292, H3122 and PC-9 lung cancer cells expressing LKB1, with statistically significant increases observed in H292 and H3122 cells (Figure 4A,B). In contrast, cells lacking LKB1 (H460, A549, 11-18 and HeLa), did not have detectable changes in phospho-AMPKα (Thr172) and phospho-ACC (Ser79) (Figure 4A,B).

**Figure 5:**
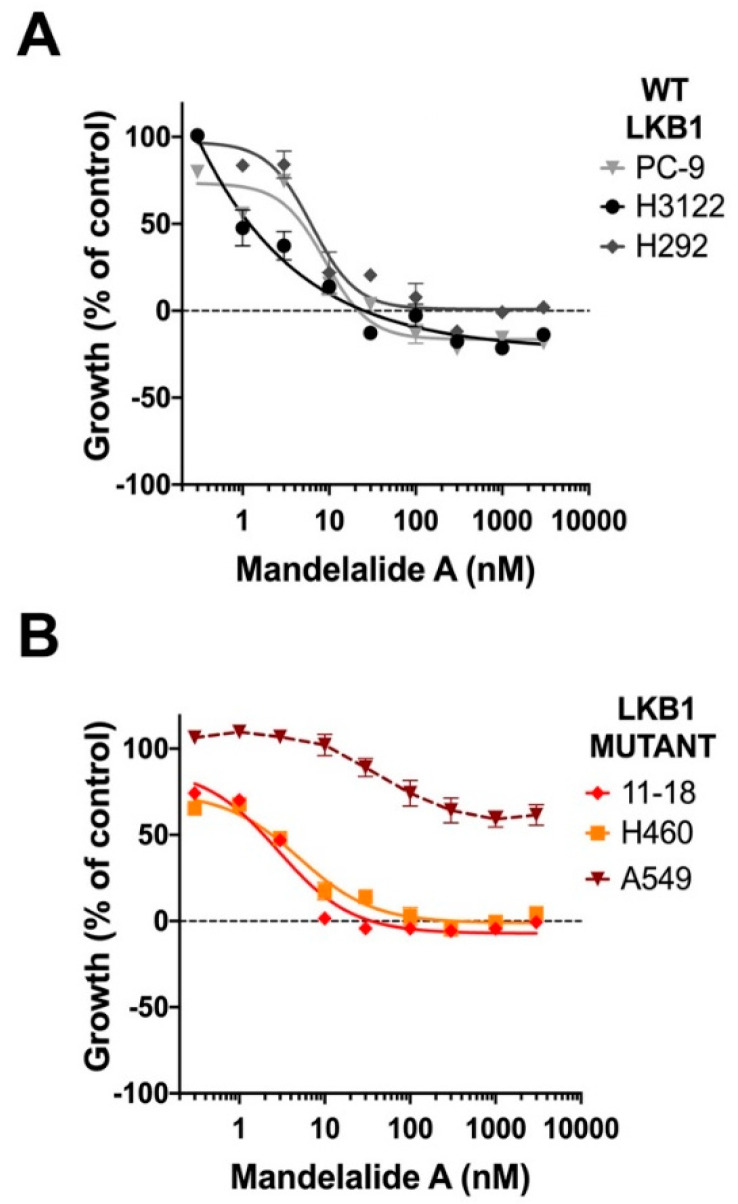
Antiproliferative activity of mandelalide A against human non-small cell lung cancer (NSCLC) cells with differing LKB1 status. Concentration-dependent changes in the growth of human NSCLC cells (**A**) expressing LKB1 (PC-9, H3122, H292) or (**B**) devoid of LKB1 (11-18, NCI-H460, A549) in response to mandelalide A or vehicle (0.1% DMSO). Antiproliferative effects were calculated using an MTT antiproliferative/viability assay with the viability of vehicle-treated cells defined as 100%. Cells were either untreated (time zero) or exposed to treatment for 72 h. Cell density at the time of treatment is indicated by a dashed black line at 0% growth. Graphs represent mean viability ± S.E. (*n* = 3 wells per treatment) and curves represent the fit of data points by nonlinear regression analysis to a logistic equation. Curves represent the fit of data from at least three independent comparisons.

The antiproliferative activity of mandelalide A was also compared across the same NSCLC cell types. NCI-H460, H292, H3122, A549, 11-18 and PC-9 lung cancer cells were treated in parallel with increasing concentrations of mandelalide A (0.1 nM to 3 μM) for 72 h. By recording cell density at the start of mandelalide treatment (t = zero), we distinguished antiproliferative from cytotoxic responses. These studies demonstrated no significant difference in the antiproliferative activity of mandelalide A against the NSCLC cells tested that could be attributed to LKB1 status (Figure 5; Table 2). Mandelalide A was a potent antiproliferative agent to NSCLC cells with wild-type LKB1 (H3122, H292, PC-9) and two of the cell lines lacking LKB1 (11-18 and NCI-H460) with GI_50_ values in the nanomolar range (Figure 5A,B). LKB1-null A549 cells remained relatively resistant to mandelalide A under these assay conditions (Figure 5B). These results indicate that mandelalide A stimulates the LKB1-AMPK signaling pathway in response to energy stress; however, the presence of LKB1 does not provide any significant protection against the antiproliferative effects of mandelalide at 72 h (Table 2).

### 2.4. Analysis of Mandelalide-Induced Cytotoxicity in EGFR Mutant NSCLC Cells Alone and in Combination with Erlotinib

**Figure 6:**
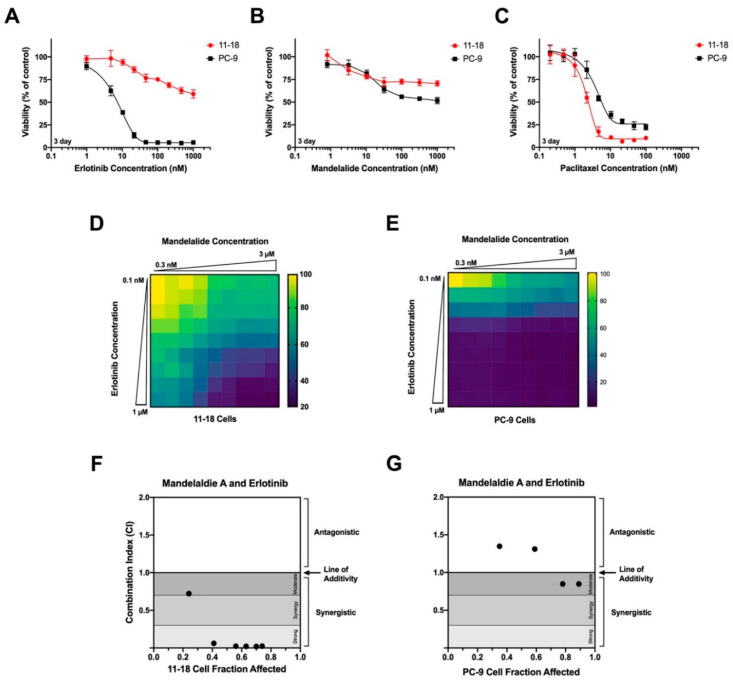
Analysis of the cytotoxic efficacy of mandelalide A in combination with erlotinib. Concentration-dependent changes in the viability of human 11-18 and PC-9 NSCLC cells in response to (**A**) erlotinib, (**B**) mandelalide A or (**C**) paclitaxel alone. Cells were exposed to increasing concentrations of each compound, as indicated, and cell viability was determined at 72 h with the viability of vehicle-treated (0.1% DMSO) cells defined as 100% viability. Data points represent mean viability ± S.E. (*n* = 3 wells per treatment) and curves represent the fit of data points by nonlinear regression analysis to a logistic equation. Cell viability curves are representative of a comparison that was repeated three times. (**D**,**E**) Heat map representing relative cell viability (%) in response to mandelalide A and erlotinib in combination. Heat maps represent representative experiments for (**D**) 11-18 and (**E**) PC-9 cells for a combination matrix experiment that was repeated three times with similar results. Combination index plots for (**F**) 11-18 and (**G**) PC-9 NSCLC cells using the Chou-Talay Combination Index method. Points below 1 indicate synergy.

Of the six NSCLC cell lines tested, two have clinically-relevant activating mutations in epidermal growth factor receptor (EGFR), and were selected for further analysis. Human 11-18 cells harbor a point (L848R) mutation, while PC-9 cells have an EGFR deletion from Glu746 to Ala750 [30]. For these studies, low-density cultures were treated with increasing concentrations of mandelalide A (1 nM to 1 μM), the receptor tyrosine kinase inhibitor (TKI) erlotinib (1 nM to 1 μM), or paclitaxel (0.2 nM to 100 nM), and cell viability was assessed in all plates at 72 h relative to vehicle (0.1% DMSO)-treated cells. Comparative analysis of these concentration-response data revealed a clear differential response of these cell types to erlotinib; PC-9 cells were highly responsive, whereas 11-18 cells were relatively resistant (Figure 6A; Table 3). As anticipated from earlier testing [3], mandelalide A alone showed limited cytotoxic efficacy to 11-18 and PC-9 cells under these assay conditions (Figure 6B; Table 3), whereas both cell types were sensitive to the microtubule-targeting agent paclitaxel (Figure 6C, Table 3).

Human NSCLC cell lines harboring mutant EGFR were exposed to increasing concentrations of mandelalide A, erlotinib, paclitaxel or vehicle (0.1% DMSO), and the metabolic activity of the cultures was assessed at 72 h using an MTT assay. Relative IC_50_ values were determined by nonlinear regression analysis fit of the data to a logistic equation, with the viability of vehicle-treated control cells was used to define 100% viability. Values represent the average of three independent experiments.

The potential for mandelalide A to modulate the action of erlotinib in both of these cell types was tested by co-treating 11-18 or PC-9 NSCLC cells with mandelalide A and erlotinib using a dose-response combination matrix. Cell viability was then assessed at 72 h relative to control cells treated with vehicle (0.1% DMSO) alone. These assays revealed a significant reduction in 11-18 cell viability in response to mandelalide A plus erlotinib that was greater than the response to either compound alone (Figure 6D). The combination of mandelalide A and erlotinib also decreased PC-9 cell viability, however this response was evident only at lower erlotinib concentrations; erlotinib alone showed full cytotoxic efficacy against PC-9 cells and was more effective than paclitaxel (Figure 6E). Computational analysis of these pharmacological data utilizing the Chou-Talalay Combination Index method revealed a strong synergistic effect of the combination of mandelalide A and erlotinib against 11-18 cells (Figure 6F) but not against PC-9 cells (Figure 6G). These data indicate that mandelalide A can enhance the effect of a clinically-relevant therapeutic TKI with cell-type specificity.

### 2.5. Mandelalide A-Induced Loss of ATP Is Lethal to Cultured Human Glioblastoma Cells

**Figure 7:**
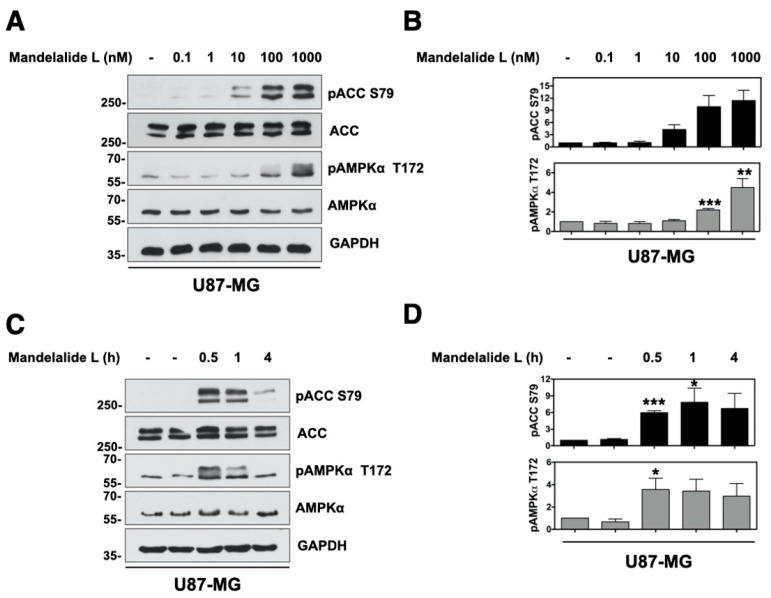
Mandelalide L induces time and concentration-dependent phosphorylation of AMPK and ACC. (**A**) Immunoblot analysis of U87-MG glioblastoma cells treated with mandelalide A for 30 min. Whole cell lysates were probed with antibodies for phospho-AMPK, phospho-ACC, total AMPK, total ACC and GAPDH as indicated. Blots are representative of a single experiment that was repeated three times. (**B**) Histograms show quantification of immunoblot data shown in A from three independent experiments; phospho-AMPK/total AMPK and phospho-ACC/total ACC, normalized to loading control (**C**) Immunoblot analysis of U87-MG treated with 1 μM of mandelalide L for 30 min, 1 h or 4 h and probed with antibodies shown in A. (**D**) Histograms show the quantification of immunoblot data shown in C from three independent experiments; phospho-AMPK/total AMPK and phospho-ACC/total ACC, normalized to loading control. Statistical significance of change relative to vehicle (0.1% DMSO) is indicated in B and D as * *p* < 0.05, ** *p* < 0.01, *** *p* < 0.001.

**Figure A1:**
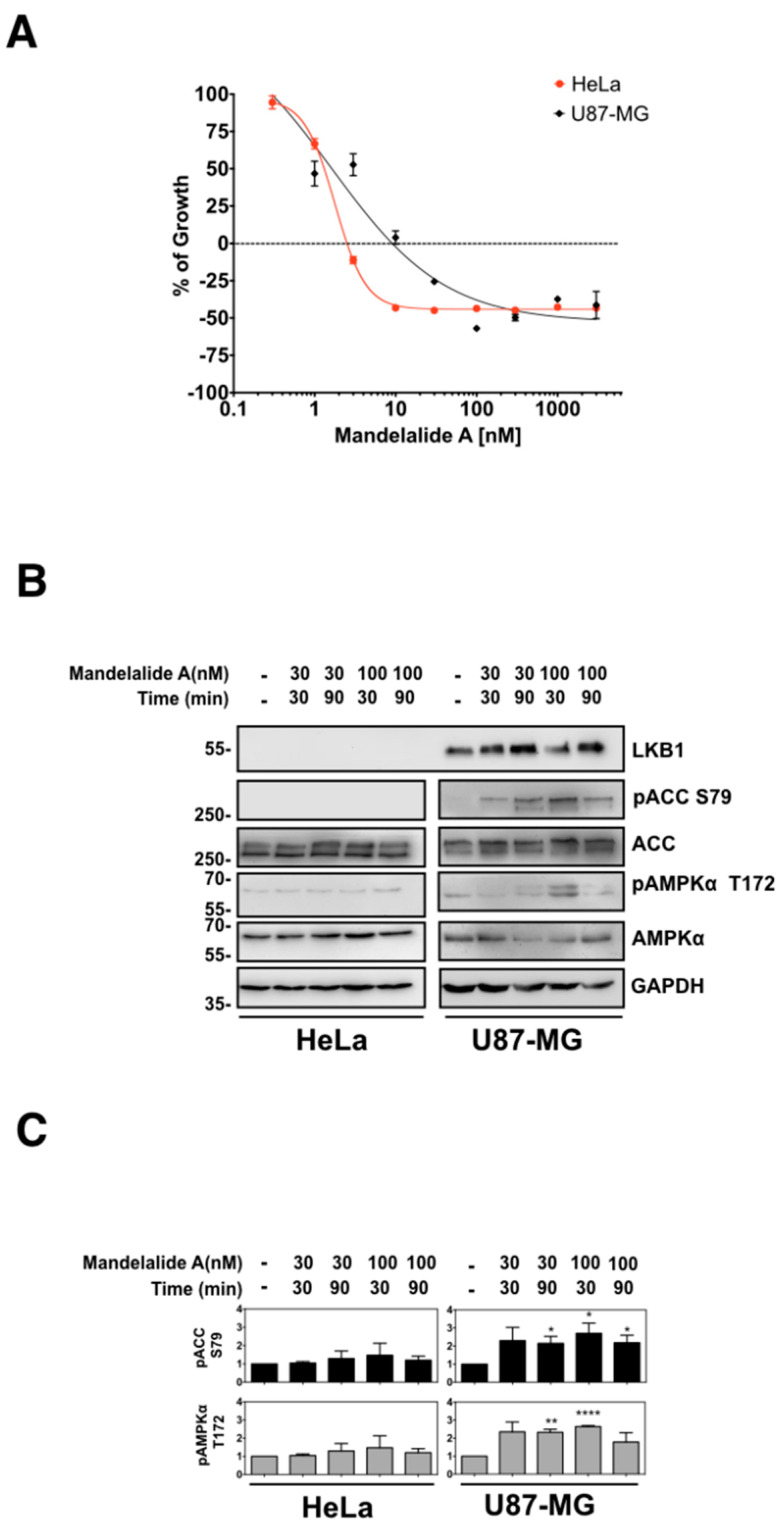
Comparative analysis of mandelalide A-induced changes in growth and AMPK signaling in human HeLa cervical and U87-MG glioblastoma cells. (**A**) Concentration-dependent changes in the growth of human HeLa cells and U87-MG glioblastoma cells in response to mandelalide A or vehicle (0.1% DMSO). Antiproliferative effects were calculated using an MTT antiproliferative/viability assay with the viability of vehicle-treated cells defined as 100%. Cells were either untreated (time zero) or exposed to treatment for 72 h. Cell density at the time of treatment is indicated by a dashed black line at 0% growth. Graphs represent mean viability ± S.E. (*n* = 3 wells per treatment) and curves represent the fit of data points by nonlinear regression analysis to a logistic equation. Curves represent the fit of data from at least three independent comparisons. (**B**) Immunoblot analysis of AMPK and ACC status in HeLa cervical and U87-MG glioblastoma cells treated with (+) mandelalide A (30 nM or 100 nM) or (−) vehicle (0.1% DMSO) for 30 min or 90 min. Whole cell lysates were collected, processed for western blot and probed with primary antibodies against phospho-AMPK, phospho-ACC, total AMPK, total ACC, LKB1 and GAPDH as indicated. (**C**) Histograms show the quantification of phospho-AMPK/total AMPK and phospho-ACC/total ACC normalized to GAPDH from three independent experiments. Statistical significance of change relative to vehicle (0.1% DMSO) is indicated as * *p* < 0.05, ** *p* < 0.01, **** *p* < 0.0001.

We selected human glioblastoma cells as a second histological cancer cell type for further evaluation of mandelalide action since these tumors have complex metabolic characteristics and remain largely resistant to current standards of care [31]. The potential of natural mandelalide L to induce an AMPK stress response was first analyzed in U87-MG glioblastoma cells, as mandelalide L has an A-type macrocycle and was previously found to be more toxic to human HeLa cervical and NCI-H460 lung cancer cells than mandelalide A [4]. For these studies, U87-MG cells were treated with, or without, increasing concentrations of mandelalide L (0.1 nM or 1 µM) for 30 min, or with a fixed concentration of mandelalide L (1 µM) for up to 4 h, and whole cell lysates were prepared for immunoblot analysis. Treatment with mandelalide L induced increases in the phosphorylation status of AMPKα (Thr172) and ACC (Ser79) relative to control cells treated with vehicle (0.1% DMSO) alone, in both a concentration- (Figure 7A) and time-dependent (Figure 7B) manner. The activation of AMPK by mandelalide L appeared to be transient, with no statistically significant increase in phospho-AMPKα (Thr172) or phospho-ACC (Ser79) at 4 h. Short-term exposure of U87-MG cells to nanomolar concentrations of mandelalide A induced the same AMPK response pattern (Figure A1) and matched that seen for mandelalide A in wild-type MEFs (Figure 2A,B).

**Figure 8:**
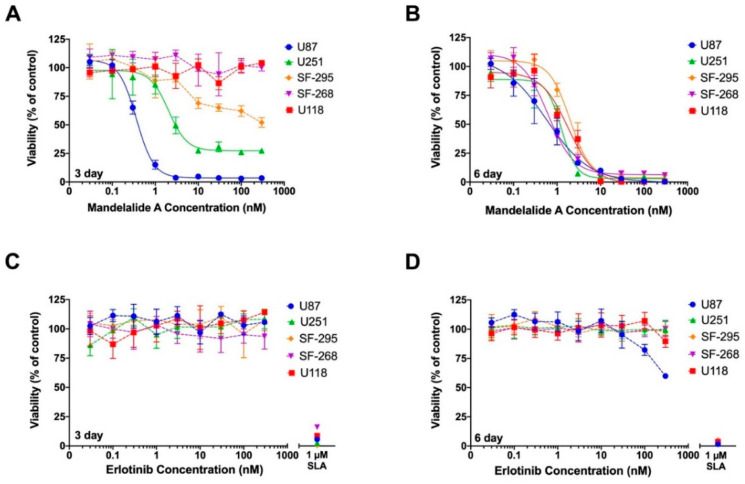
Mandelalide A is a potent and efficacious cytotoxin to human glioblastoma cells. Concentration-response relationships for (**A**,**B**) mandelalide A- or (**C**,**D**) erlotinib-induced toxicity to human U87-MG, U251, F-295, SF268 and U118 glioblastoma cells. Cells were continuously exposed to test compound or vehicle (0.1% DMSO) for three days (**A**,**C**) or six (three + three) days (**B**,**D**). Cell viability was assessed using a CellTiter-Glo^®^ assay with the viability of vehicle-treated control cells defined as 100%. A single concentration of salinosporamide A (SLA; 1 µM) was used as a positive cytotoxic control. Graphs represent mean viability ±S.E. (*n* = three wells per treatment) and curves represent the fit of data points by nonlinear regression analysis to a logistic equation. Curves represent the fit of data from at least three independent comparisons.

To gain a better understanding of the inherent resistance of some cancer cell types to the mandelalides, we tested the cytotoxic potential of mandelalide A against a panel of validated glioblastoma cell lines. Using an assay technique that we previously applied to study the action of apoptolidin A [25], human U87-MG, U251, SF-295, SF-268 and U118 glioblastoma cells were treated in parallel with increasing concentrations of mandelalide A (0.03 nM to 300 nM), erlotinib (0.03 nM to 300 nM), or vehicle (0.1% DMSO) for three days or six days. For six day end point studies, a culture medium containing the same treatment was replaced at three days to avoid nutrient deprivation (termed three days + three days) [25]. The proteasome inhibitor salinosporamide A (1 µM) was used as an independent control to define 100% cell kill and, at the designated end point, cell viability was assessed by direct quantification of ATP. As anticipated from the NCI60 CNS cancer panel screening results, which utilize a 48 h end point [4], glioblastoma cells showed highly variable responses to mandelalide A after three days (Figure 8A). U87-MG cells were most sensitive (EC_50_ = 0.38 nM), U251 and SF295 were somewhat sensitive, whereas SF-286 and U118 cells were fully resistant to the highest mandelalide A (300 nM) concentration tested (Figure 8A; Table 4). When exposure time was increased to six days, however, mandelalide A showed low nanomolar potency and full cytotoxic efficacy against all five glioblastoma cell types (Figure 8B; Table 4). Most cells were inherently resistant to erlotinib (Figure 8C,D; Table 4), with only U87-MG cells showing a reduction in viability at higher concentrations after six days (Figure 8D). These results provide insight into the metabolic flexibility of glioblastoma cells and suggest that the major cytotoxic mandelalides (A, B and L) induce an early AMPK stress response in glioblastoma cells, which is overwhelmed with prolonged exposure.

## 3. Discussion

Numerous natural products from terrestrial plants, fungi and bacteria have been shown to activate AMPK in mammalian cells in response to the site-specific inhibition of mitochondrial function [32]. It has also been noted that at lower, sub-toxic doses, many of these plant-derived activators of AMPK have a long history of use as traditional herbal medicine and as reference chemicals that continue to inspire the development of pharmacological activators of AMPK for the treatment of conditions such as insulin resistance, inflammation, metabolic disease and cancer [32,33]. The results of the present study indicate that A-type mandelalides, and presumably mandelalide B, induce a pattern of indirect AMPK activation. This response to the ATP-depleting mandelalides is in agreement with the well-established physiological role of AMPK as a central metabolic regulator that acts to maintain cellular ATP and nucleotide balance in response to energy stress [34,35]. This consequence of mandelalide action in cells is also consistent with that observed for other plant and microbial secondary metabolites that are known to interact directly with either mitochondrial complex I of the respiratory electron transport chain or the ATP synthase complex V [33,36,37]. These findings complement earlier metagenomic sequence analyses of field-collected material by the Kwan laboratory and the conclusion that the mandelalides are the product of an ancient bacterial symbiont that has likely evolved as a cytotoxic chemical defense for the tunicate host [5]. Although the present study did not test the consequences of mandelalide exposure in an ecological context of natural predation of the marine tunicate *Lissoclinum* sp., we report that the toxic, ATP-depleting members of the mandelalide family of macrolactones [4], induce AMPK-mediated survival signaling in intact mammalian cells.

Early evaluation of the biological activity of the natural mandelalides had revealed a sensitivity of at least two LKB1-null human cancer cells (HeLa cervical and NCI-H460 NSCLCs [1,9]), leading us to investigate LKB1 status as a potential determinant of mandelalide sensitivity. LKB1 is the major upstream kinase required for activation of AMPK, and other related kinases, in response to energy stress [29,38,39]. The discovery of the LKB1-AMPK signaling pathway represented a major breakthrough in the study of cancer metabolism given that mutations in LKB1 had previously been identified in patients with Peutz-Jeghers syndrome, which is associated with benign growths and increased risk of a range of different cancers [29,40,41]. LKB1 is now recognized as a critical node in the regulation of cellular growth and metabolism, with inactivation or deletion of LKB1 identified in a significant percentage of human cancers, including patients with NSCLC and cervical cancer [42]. In the present study, mandelalide A-induced phosphorylation of AMPKα (Thr172) was detected only in the LKB1-positive NSCLC cell types, however LKB1 status alone failed to predict the sensitivity of cells to the growth inhibitory effects of mandelalide A. GI_50_ values for five of the six NSCLC cell types tested were in the low nanomolar range, with no significant difference between LKB1-positive cells (H292, H3122 and PC-9) and LKB1-null H460 or 11-18 NSCLC cells. However, when EGFR status was considered, mandelalide A in combination with erlotinib showed promise against 11-18 NSCLC cells that were relatively resistant to erlotinib alone. EGFR-targeted therapies are now considered to be a first-line treatment for NSCLC with second and third generation EGFR inhibitors in use as a strategy to overcome the major clinical complication of resistance to TKIs [43]. It is also recognized that an additional barrier to treatment can occur when EGFR inhibitors induce a reverse Warburg effect whereby OXPHOS is upregulated as a favored bioenergetic survival strategy [44]. This observation has, in turn, raised the possibility that OXPHOS could be targeted in TKI-resistant NSCLC, which is proposed to be the mechanistic basis for the effectiveness of the mitochondrial complex I inhibitors metformin and phenformin in a variety of preclinical lung cancer models [45,46,47]. Phenformin was previously found to have superior efficacy against LKB1-deficient NSCLC cells [48], and thus our observation that the action of mandelalide A may be independent of LKB1 status provides a starting point for further investigation of ATP synthase inhibitors against TKI-resistant NSCLC cell types. Although the precise mandelalide-ATP synthase protein complex binding site is not known, structural evidence has demonstrated that differences in the way natural product mitochondrial ATP synthase inhibitors bind the same target can provide new drug leads against OXPHOS-dependent cancers [21,49]. For example, other glycosylated macrolactones, such as apoptolidin A and ammocidin A, bind the F_1_ portion of ATP synthase to inhibit mitochondrial function in a fundamentally different way than the F_0_-binder oligomycin A [50], with ammocidin A recently emerging as a more promising preclinical lead for OXPHOS-dependent leukemia [49].

Mandelalides A and L tended to induce early AMPK activation that plateaued within hours of exposure and was reminiscent of a pattern of resistance to AMPK activation that has been observed with other chemical inhibitors. For example, studies of OXPHOS (oligomycin A) or glycolysis (2-deoxyglucose) inhibitors alone, and in combination, showed that MEFs can compensate for the presence of a single metabolic inhibitor, and this effectively confers resistance to AMPK activation [51]. Similarly, inhibition of complex I (metformin) or glycolysis (2-deoxyglucose) alone resulted in only moderate activation of AMPK, whereas inhibition of both metabolic pathways resulted in greater activation of AMPK in prostate cancer cells and enhanced apoptosis when metformin and 2-deoxyglucose were combined as an anticancer treatment [52,53]. Although we did not test AMPK activation in response to the combination of mandelalide A and 2-deoxyglucose, we have previously shown that the cytotoxic potential of mandelalide A is heavily influenced by the underlying metabolic phenotype of the cell. HeLa cervical cancer cells grown at low density were more sensitive to 2-deoxyglucose than mandelalide A, whereas high-density cultures, with a more oxidative phenotype, were sensitive to mandelalide A and relatively resistant to 2-deoxyglucose [3]. Consistent with the observations of Ben Sahara et al. that prostate cancer cells were more sensitive to the combination of metformin and 2-deoxyglucose [53], the combination of mandelalide A and 2-deoxyglucose was also highly toxic to HeLa cervical cancer cells [3]. Thus, the ability of mandelalide A alone to induce an AMPK stress response is also likely to be influenced by both basal metabolic phenotype and metabolic flexibility of the cell type.

Since the discovery of oncogenic mutations that affect the Krebs cycle, metabolic adaptation has been increasingly recognized as an important molecular feature of glioblastoma multiforme (GBM) [54,55]. Many preclinical and clinical studies have consequently been designed to target pathways that support cellular respiration, and include the study of known and novel small inhibitors of OXPHOS or mitochondrial function [18,56,57]. AMPK has also been proposed as a relevant and druggable therapeutic target for the treatment of brain tumors [58]. Specific isoforms of AMPK subunits, including α1, are highly expressed in patients with GBM relative to expression levels found in normal human brain or those with low grade glioma [59]. These changes in expression level are not prognostic but appear to have functional significance; AMPK activation represents a chronic stress adaptation that confers a survival advantage by inducing cellular proliferation and cell cycle progression that is particularly advantageous for the survival of primary glioma stem cells (GSCs) [58,59]. In the present study we confirmed the time-dependent nature of mandelalide-induced cytoxicity to individual GBM cell types and the high sensitivity of U87-MG glioblastoma cells to cytotoxic mandelalides [3,4]. As predicted from earlier studies with living cells in which SF-295 cells were found to have a relatively glycolytic metabolic phenotype when exposed to apoptolidin A [25], mandelaide A was a less potent and efficacious cytotoxin to SF-295, SF-268 and U118 glioblastoma cells when assays were terminated at 72 h. In contrast, U87-MG glioblastoma cells, which are much more dependent upon OXPHOS as a bioenergetic strategy [25], displayed clear changes in AMPK activation in response to the depletion of cellular ATP by mandelalide L. All compensatory survival signaling in these cells could be overwhelmed by prolonged exposure to mandelalide A, suggesting that the cytotoxic mandelalides are a potentially useful class of ATP synthase inhibitors to probe metabolic flexibility in glioblastoma cells and GSCs relative to astrocytes and other normal cell types.

## 4. Materials and Methods

### 4.1. Chemicals and Reagents

The recollection and isolation of natural product mandelalides from Algoa Bay, South Africa and total syntheses of mandelalides A and L have been described previously [4,9,60]. NMR data for mandelalides tested here are included in [4] (natural products) and 61 (synthetic products), and were acquired at the start of this study; integrity of all mandelalide compounds was also routinely confirmed by LC-MS. Oligomycin A was purchased from Santa Cruz Biotech (Dallas, TX, USA). Dorsomorphin was purchased from Abcam (Cambridge, UK). Paclitaxel and salinosporamide A (marizomib) were purchased from Sigma Aldrich (St. Louis, MO, USA). Erlotinib was purchased from Enzo Life Sciences (Farmingdale, NY, USA). All chemicals were reconstituted in 100% DMSO, aliquoted and stored in amber borosilicate glass vials at −20 °C for use in biological studies. Cell culture-grade DMSO was used as the vehicle for all treatments, and final concentrations of DMSO for in vitro experiments never exceeded 0.1%. Primary and secondary antibodies were from Cell Signaling Technology, Inc. (Danvers, MA, USA). Specific codes were as follows: AMPK (#5831), pAMPKα T172 (#50081), ACC (#3676), pACC S79 (#11818) and GAPDH (#5174). General laboratory reagents were from VWR International (Radnor, PA, USA).

### 4.2. Mammalian Cell Culture

Immortalized AMPKα double-knockout MEFs were generated by crossing AMPKα1^−/−^ and AMPKα2^−/−^ mice for isolation of fibroblasts, culture and verification by sequencing [61]. Human U87-MG glioblastoma, U118 glioblastoma, HeLa cervical, H292, A549 and H3122 lung cancer cells were from the American Type Culture Collection (Manassas, VA, USA). Human SF-295, SF-268 and U251 glioblastoma cells and NCI-H460 lung cancer cells were obtained from the National Cancer Institute (NCI) cell line repository (Fredrick, MD, USA). Human PC-9 and 11-18 lung cancer cells were a kind gift from Dr. Monkia Davare (Oregon Health & Science University, Portland, OR, USA). MEFs were cultured in Dulbecco’s Modified Eagle’s Medium (Corning^®^ DMEM, Life Sciences, Durham, NC, USA) with 10% FBS, 1% penicillin and streptomycin. U87-MG, U251 and HeLa cells were cultured in Minimum Essential Medium (MEM) with Earl’s salts and L-glutamine (Corning Life Sciences), supplemented with 10% fetal bovine serum (FBS; Hyclone, Logan, UT, USA), and 100 U/mL penicillin and 100 mg/mL streptomycin (1% penicillin/streptomycin). U118 and SF-268 cells were cultured in Dulbecco’s Modified Eagle’s Medium (DMEM) with 10% FBS, 1% penicillin/streptomycin, L-glutamine (6 mM), and sodium pyruvate (1 mM). SF-295 cells were cultured in RPMI 1640 medium with 10% FBS, and 1% penicillin/streptomycin. NCI-H460, H292, H3122, 11-18 and PC-9 cells were cultured in RPMI-1640 (Sigma Aldrich) supplemented with 10% FBS and 1% penicillin and streptomycin. A549 cells were cultured in F-12K medium (Thermo Fisher Scientific Inc., Waltham, CA, USA) with 10% FBS, 1% penicillin and streptomycin. All cells were maintained as adherent cultures under standard conditions and maintained at 37 °C in an atmosphere of 5% CO_2_.

### 4.3. Cell Lysis and Immunoblot Analysis

Cell lysates were prepared using a freshly prepared ice-cold Lysis Buffer containing 50 nM Tris-HCl (PH 7.5), 1 mM EDTA, 1 mM EGTA, 1% Triton X-100, 0.27 M Sucrose, 50 mM sodium fluoride, 1 mM sodium orthovanadate, 5 mM sodium pyrophosphate, 1 mM PMSF and 1 mM benzamidine. All cell lysates were cleared by centrifugation at 16,000× *g* for 20 min at 4 °C and the protein concentration was determined by the bicinchoninic acid (BCA) method indicated by the manufacturer’s recommendations (Thermo Fisher Scientific, Waltham, MA, USA).

For immunoblot analysis, cell lysates were adjusted by protein concentration and equal amounts (50 μg) separated by SDS-PAGE gel. Proteins were then transferred onto a PVDF membrane (Thermo Fisher Scientific, Waltham, MA, USA) in a transfer buffer containing 25 nM Tris, 192 mM glycine, and 10% methanol. Membranes were then blocked in 5% (*m*/*v*) non-fat milk in 50 mM Tris-HCl, pH 7.4, 150 mM NaCl (TBS) with 0.05% Tween-20 (TBS-T), and incubated at 4 °C for 16 h with appropriate primary antibodies in 5% (*w*/*v*) bovine serum albumin (BSA) in TBS-T. On the following day, membranes were washed with TBS-T for 2 × 5 min, then incubated in appropriate HRP-conjugated secondary antibodies for 1 h at room temperature. Membranes were then washed in TBS-T for 3 × 5 min, and target proteins were detected by chemiluminescence (Amersham^TM^ ECL^TM^ Chemiluminescent Labeling and detection Reagents for Proteins, GE Healthcare, Chicago, IL, USA), using myECL^TM^ Imager system (Thermo Fisher Scientific, Waltham, MA, USA).

### 4.4. Cell Viability Assay

Cells were seeded at a density of 3000 cells/well in 100 µL of complete medium in 96-well plates. After 18 h, cells were treated with mandelalide A or vehicle (0.1% DMSO), or processed as a time zero, untreated plate. Cell viability was assessed in all treated plates at the assay endpoint using a CellTiter-Glo^®^ Luminescent Cell Viability Assay kit (Promega Corp., Madison, WI, USA) or standard 3-(4,5-dimethylthiazol-2-yl)-2,5-diphenyltetrazolium bromide (MTT) assays, with the viability of vehicle-treated cells defined as 100%. For long exposures (three + three day), assays were performed as described previously [25]. Briefly, after three days the cell culture medium was replaced with fresh medium and maintained under identical treatment conditions for the remaining three days of treatment.

### 4.5. Drug Combination Assays

Human 11-18 and PC9 lung cancer cells were seeded at a density of 1000 cells/well in 384-well white-wall solid flat bottom plates (Greiner Bio-One, Frickenhausen, Germany) with 50 µL of complete medium. After 18 h, cells were treated with vehicle (0.1% DMSO) or titration of mandelalide A in combination with erlotinib. Treatment was completed by a Hewlett-Packard (HP) Tecan D300 Digital dispenser (HP, Palo Alto, CA, USA). After 72 h of treatment, cell viability was assessed using a CellTiter-Glo^®^ Luminescent Cell Viability Assay kit (Progema Corp., Madison, WI, USA).

### 4.6. Data Analysis

Concentration-response relationships were analyzed using Graphpad Prism Software version 8.0 (Graphpad Software Inc., San Diego, CA, USA). GI_50_, TGI or LC_50_ values were determined by nonlinear regression analysis fit to a logistic equation using Graphpad Prism Software (La Jolla, CA, USA). For immunoblot analysis, target phospho-protein signals were calculated relative to total protein and normalized to the intensity of control proteins (tubulin or GAPDH), using ImageJ software (rsbweb.nih.gov/ij (accessed on 1 November 2021)). Statistical significance of data derived from cell viability assays and quantification of immunoblot assays were performed on Graphpad Prism Software using a one-way analysis of variance (ANOVA) followed by a Student’s *t*-test comparing untreated controls and treatment groups. *p*-Values of 0.05 or less were considered statistically significant. The potential synergy between mandelalide A and erlotinib was evaluated using the Chou-Talay Combination Index method [62,63,64] and CompuSyn software (ComboSyn, Inc., Paramus, NJ, USA).

## 5. Conclusions

The results of the present study serve to add members of the mandelalide natural product series of polyketide macrolactones to a large diverse group of secondary metabolites that are also known pharmacological activators of AMPK. Specifically, the bioactivity profile of glycosylated mandelalides A, B and L is consistent with compounds that are designated as indirect pharmacological AMPK activators. These complex macrocyclic structures effectively deplete cellular ATP in living cells and induce a concomitant increase in AMP and ADP, activating the AMPK enzyme to restore energy homeostasis [4,36]. Although AMPK activators are well represented in terrestrial systems [36], the marine environment has a relatively short history of exploration as a source of new molecules, with the ecology of symbiotic marine-living verrucomicrobial microbes even less studied [5,65]. Taken together, these results expand the chemical diversity of AMPK activators and illustrate the range of unique habitats in which this highly conserved signal transduction system exists. With synthetic routes now optimized for the A-type mandelalides and several potent non-natural analogues identified [60], these results provide the basis for future analysis of the biological activity of mitochondrial ATP synthase and AMPK activation in mandelalide-sensitive cell types.

## Figures and Tables

**Table 1:** Relative cytotoxic potencies of mandelalide A to wild-type and AMPKα-null mouse embryonic fibroblasts.

	Relative IC_50_ (nM) ± SEM		
	24 h	48 h	72 h
Wild-type	28.2 ± 6.3	37.1 ± 0.1	33.0 ± 4.9
AMPKα-null	9.3 ± 1.3	13.5 ± 1.4 **	10.6 ± 1.9 *

Mouse embryonic fibroblasts were exposed to increasing concentrations of mandelalide A, or vehicle (0.1% DMSO), and the metabolic activity of the cultures was assessed at three different endpoints using an MTT assay. Relative IC_50_ values were determined by nonlinear regression analysis fit to a logistic equation, with the viability of vehicle-treated control cells used to define 100% viability. Values represent the average of three independent experiments. Statistical significance between IC_50_ values was compared for wild-type and AMPKα-null cells using an unpaired *t*-test and is indicated as * *p* < 0.05, ** *p* < 0.005.

**Table 2:** Comparative analysis of the antiproliferative activity of mandelalide A to a panel of human non-small cell lung cancer (NSCLC) cells.

Cell Line	LKB1 Status	EGFR Status	GI_50_ (nM) ± SEM	TGI (nM) ± SEM
11-18	Loss	L848R	2.0 ± 0.4	174 ± 26
H460	Loss	WT	4.3 ± 0.6	50.2 ± 8.9
A549	Loss	WT	~300	>3000
H3122	WT	WT	4.7 ± 0.6	45.8 ± 13
H292	WT	WT	3.4 ± 0.2	51.5 ± 12
PC-9	WT	E746- A750 del	3.6 ± 0.7	133 ± 46

Human NSCLC cell lines with differing LKB1 and EGFR status were exposed to increasing concentrations of mandelalide A, or vehicle (0.1% DMSO), and the metabolic activity of the cultures was assessed at 72 h using an MTT assay. GI_50_ and TGI values were determined by nonlinear regression analysis fit of the data to a logistic equation, with the viability of vehicle-treated control cells used to define 100% viability. Untreated cells assayed at the start of the study were used to define time = 0. Values represent the average of three independent experiments.

**Table 3:** Relative cytotoxic potencies of mandelalide A, erlotinib or paclitaxel to human non-small cell lung cancer (NSCLC) cells with clinically-relevant EGFR mutations.

	Relative IC_50_ (nM) ± SEM		
	Mandelalide A	Erlotinib	Paclitaxel
11-18	~100 ^1^	~100 ^1^	2.94 ± 0.80
PC-9	~100 ^1^	5.24 ± 2.35	5.20 ± 2.13

^1^ Limited cytotoxic efficacy; absolute IC_50_ not determined (>1000 nM).

**Table 4:** Comparative analysis of the cytotoxic potential of mandelalide A or erlotinib to a panel of human glioblastoma cells.

	Relative IC_50_ (nM) ± SEM			
Cell Line	Mandelalide A 3 Day	Mandelalide A 6 Day	Erlotinib 3 Day	Erlotinib 6 Day
U87-MG	0.38 ± 0.01	0.85 ± 0.11	>300	~100 ^1^
U251	1.72 ± 0.22	1.21 ± 0.26	>300	>300
SF-295	>10 ^1^	1.26 ± 0.23	>300	>300
SF-268	>300	1.07 ± 0.26	>300	>300
U118-MG	>300	1.21 ± 0.19	>300	>300

^1^ Limited cytotoxic efficacy; absolute IC_50_ not determined (>300 nM or not active). Human glioblastoma cells were exposed to increasing concentrations of mandelalide A, erlotinib, or vehicle (0.1% DMSO), and the metabolic activity of the cultures was assessed at three days or six (three + three) days using a CellTiter-Glo^®^ assay. Relative IC_50_ values were determined by nonlinear regression analysis fit of the data to a logistic equation, with the viability of vehicle-treated control cells used to define 100% viability. A single concentration of salinosporamide A (1 µM) was used as a positive cytotoxic control. Values represent the average of three independent experiments.
